# Pressure and high-T_c_ superconductivity in sulfur hydrides

**DOI:** 10.1038/srep25608

**Published:** 2016-05-11

**Authors:** Lev P. Gor’kov, Vladimir Z. Kresin

**Affiliations:** 1 NHMFL, Florida State University, 1800 East Paul Dirac Drive, Tallahassee, Florida 32310, USA; 2 L.D. Landau Institute for Theoretical Physics of the RAS, Chernogolovka 142432, Russia; 3 Lawrence Berkeley Laboratory, University of California, 1 Cyclotron Road, Berkeley, CA 94720, USA

## Abstract

The paper discusses fundamentals of record-*T*_*C*_ superconductivity discovered under high pressure in sulfur hydride. The rapid increase of *T*_*C*_ with pressure in the vicinity of *P*_*cr*_ ≈ 123*GPa* is interpreted as the fingerprint of a first-order structural transition. Based on the cubic symmetry of the high-*T*_*C*_ phase, it is argued that the lower-*T*_*C*_ phase has a different periodicity, possibly related to an instability with a commensurate structural vector. In addition to the acoustic branches, the phonon spectrum of H_3_S contains hydrogen modes with much higher frequencies. Because of the complex spectrum, usual methods of calculating *T*_*C*_ are here inapplicable. A modified approach is formulated and shown to provide realistic values for *T*_*C*_ and to determine the relative contributions of optical and acoustic branches. The isotope effect (change of *T*_*C*_ upon Deuterium for Hydrogen substitution) originates from high frequency phonons and differs in the two phases. The decrease of *T*_*C*_ following its maximum in the high-*T*_*C*_ phase is a sign of intermixing with pairing at hole-like pockets which arise in the energy spectrum of the cubic phase at the structural transition. On-pockets pairing leads to the appearance of a second gap and is remarkable for its non-adiabatic regime: hydrogen mode frequencies are comparable to the Fermi energy.

The search for high-*T*_*C*_ (“room”) superconductivity at high pressure was triggered by the suggestion^1^ that one can expect high values of *T*_*C*_ in systems comprised of light atoms, including the metallic hydrogen. It is based on the fact that according to the BCS theory, the transition temperature is proportional to the frequency of phonons mediating the pairing. Half a century later, superconductivity at 190 K was claimed in sulfur hydrides under pressure *P* > 150*GPa*^2^. Recently, *T*_*C*_ ≈ 203 *K* was confirmed in H_3_S formed in the decomposition of H_2_S under pressure^3^.

The present work is mainly concerned with the peculiar pressure dependence of the superconducting transition temperature in sulfur hydride H_3_S. According to recent data^4^, the value of *T*_*C*_ ≈ 100 *K* at *P*_*cr*_ ≈ 123 *GPa* sharply increases to *T*_*C*_ ≈ 200*K* at*P*_*cr*_ ≈ 150 *GPa* as in a phase transition. In particular, once *T*_*C*_ reaches its maximum value *T*_*C*_ ≈ 200 *K* at the onset of high-*T*_*C*_ phase it begins immediately to *decrease* with further increase in pressure^3^,^4^.

We *assume* that the behavior of *T*_*C*_ in this pressure interval is a signature of a structural transition between phases with lower and higher *T*_*C*_. Moreover, we argue that the first-order phase transition is the most credible concept for the near-doubling of *T*_*C*_ in the narrow experimental pressure interval Δ*P* ≈ 25 *GPa* and discuss the factors which account for such a significant increase in *T*_*C*_. As concerns the microscopic mechanism which underlies the subsequent decrease in *T*_*C*_, it is related to the coupling between the superconducting order parameters on hole-like pockets and on the main (“large”) part of the Fermi surface.

One should appreciate the challenges for theory in describing a material under high pressure. The common goal has been to establish stoichiometry, to prove the stability of the phases emerging at metallization, to identify transformations between the phases, to reconstruct the electronic bands and the phonons spectra, all from the *first principles*. Having obtained this information, one may attempt to evaluate the temperature *T*_*C*_ of the superconducting transition. Such an analysis was carried out in^5^,^6^,^7^,^8^,^9^,^10^,^11^,^12^,^13^,^14^,^15^,^16^,^17^,^18^,^19^,^20^,^21^,^22^,^23^,^24^, including the successful prediction in^5^,^6^ of the H_3_S-stoichiometry in agreement with the *X*-ray experiment^4^. At the same time, one finds inconsistencies between different theoretical publications, manifested especially sharply in the uncertainty of predictions of the specific phase transition pressure and for the symmetry of the phases.

According to the most publications, the mechanism of superconductivity in the high-*T*_*C*_ phase is phonon mediated electron-electron pairing on the “*large*” part of the Fermi surface. Based on calculated electron and phonon spectra, the transition temperature *T*_*C*_ has been deduced numerically with the use of the Migdal-Eliashberg (ME) equations^25^,^26^. However, most of these algorithms were developed and optimized for ordinary metals. The applicability of the same methods to an analysis of the superconducting transition temperature in H_3_S is scrutinized in the next Section. We introduce a new method for the evaluation of *T*_*C*_ based on generalization of the ME approach to the case of such a complex phonon spectra. To be more specific, the ME equations are rewritten to account for the fact that the phonon contributions from the optical and the acoustic branches have different characteristic frequencies and coupling constants.

The isotopic dependence of *T*_*C*_ (i.e., its change upon the substitution of deuterium for hydrogen) turns out to be different for the two sides of the phase transition, in agreement with the experiments^3^,^4^. We conclude that the key role in the superconductivity of H_3_S^2^,^3^,^4^ is played by high frequency hydrogen modes.

As was noted above, the mechanisms of superconductivity described in^5^,^6^,^7^,^8^,^9^,^10^,^11^,^12^,^13^,^14^,^15^,^16^,^20^,^24^ assert the prevailing role of the Cooper pairing on the large part of the Fermi surface. Standing apart is a scenario^17^,^18^,^19^ in which superconductivity in high-*T*_*C*_phase is driven by the pairing on small hole-like pockets emerging at several spots of the Brillouin zone (BZ) via the Lifshitz 2.5-topological transition^27^,^28^.

Hole-like pockets in the band structure of the high-*T*_*C*_ phase were theoretically exhibited in^6^,^7^,^9^,^11^,^17^,^22^. The special role assigned to them in^17^ is owed to a van Hove (*vH*) singularity peak in the density of states (DOS) in close vicinity of the chemical potential, leading to a strong enhancement of the electron-phonon interactions. A peak in DOS is present in several band structure calculations^6^,^11^,^12^,^17^,^18^,^19^,^22^, in^24^ (see Fig. 4, Suppl. Mat.), but it lies at 0.15 ÷ 0.4 *eV* below the chemical potential. The results below are in better agreement with the idea that the main contribution to pairing is due to the interactions at a large part of the Fermi surface, with pockets playing only a supportive role.

Experiment^4^ and the theory agree upon the body centered cubic lattice for the *high-T*_*C*_phase of H_3_S; then the electronic and phonons spectrum above P = 200 GPa are found to be consistent and are taken as the basis for the further analysis. Discrepancies among theoretical treatments at *lower* pressure will be discussed below.

## Results

### Transition temperature in high-*T*_C_ phase

The energy scale typical for the large part of the Fermi surface (broad bands) is a few *eV*. At *T* = *T*_*C*_the equation for the order parameter Δ(*ω*_*n*_) is:


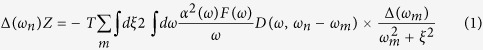


Here 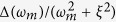 is the pairing Greens function^29^; *D*(*ω*, *ω*_*n*_ − *ω*_*m*_) = −*ω*^2^/[(*ω*_*n*_ − *ω*_*m*_)^2^ + *ω*^2^] is the phonon propagator; *ω* is the phonon frequency, ξ is the electron energy referred to the chemical potential, *ω*_*n*_ = (2*n* + 1)*πT*. We are employing the method of thermodynamic Green’s functions; see, e.g.,^30^. The function *α*^2^(*ω*)*F*(*ω*) is a well-known quantity determining the strength of the electron-phonon interaction (see, e.g.^31^,^32^), *F*(*ω*) is the phonon density of states, *Z* ≃ 1+*λ* in (1) stands for the band mass renormalization. The coupling constant *λ* is defined by the expression:


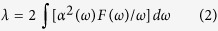


It is essential that Eq. (1) does not explicitly contain the coupling constant λ. Indeed, it involves integration over the phonon frequency *ω* which enters not only in the factor *α*^2^(*ω*)*F*(*ω*), but in the phonon propagator *D*(*ω*, *ω*_*n*_ − *ω*_*m*_) which also depends on *ω*_*n*_−*ω*_*m*_.

It is apparent from Eqs. (1, 2) that the coupling constant can be factored out if Eq. (1) does not contain a phonon propagator function (e.g., *D* ≈ 1 for the weak coupling case) or if the dependence of *D* on the frequency *ω* can be neglected.

In principle, the value of *T*_*C*_ can be calculated directly from the full non-linear equation for the order parameter Δ(*ω*_*n*_) (at *T* < *T*_*C*_ one should substitute *ξ*^2^ ⇒ *ξ*^2^ + Δ^2^ (*ω*_*n*_) in Eq. (1)). Such a program was carried out in^11^,^12^,^15^,^24^ in the framework of the superconducting density functional theory (the calculation in^24^ was extended beyond constant-DOS approximation and without treating the pseudopotential μ* as an empirical parameter). The impact of anharmonicity was studied in^15^. The value of *T*_*C*_ was calculated from the non-linear equation for Δ (*ω*_*n*_) by iterations.

An important point to emphasize is the following. The analysis of usual superconductors is based on the concept of a coupling constant, *λ* which makes it possible to obtain an analytic expression for *T*_*C*_. The fact of the matter is that in common metals the function *α*^2^(*ω*)*F*(*ω*) is characterized by a peak in the phonon density of states (DOS) *F*(*ω*) (see, e.g.^31^,^32^). This peak corresponds to the short-wavelength part of the spectrum where the mode dispersion 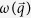 is weak. This permits the replacement of 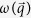 in the phonon propagator by its average value 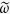
32,33,34 (the latter taken either as 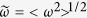, see, e.g.^33^, or 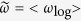, which is close to <*ω*^2>1/2^, see^35^,^36^).

The principal cause for concern about the applicability of the same scheme to H_3_S is that the phonon spectrum of sulfur hydride is complex and consists of the well-separated acoustic and optical branches; the phonon DOS contains *several* peaks. As a consequence, introducing a coupling constant *λ* and the characteristic frequency 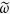 should be done with considerable care.

Our approach is to separate the phonon spectrum in the two regions of the optical and acoustic phonons and for each of them to introduce their respective average frequencies 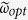 and 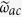 and the coupling constants *λ*_*opt*_ and *λ*_*ac*_. Such separation allows us to compare the relative contributions of the optical and acoustic phonons. Then Eq. (1) takes the following form:


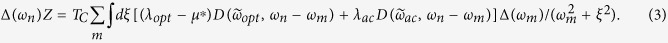


Here *λ*_*i*_ = ∫_*i*_*dωα*^2^(*ω*)*F*(*ω*)/*ω*; 
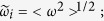
 <*ω*^2^> = (2/*λ*_*i*_)∫_*i*_*dωα*^2^(*ω*)*F*(*ω*)*ω*; *i* ≡ {*opt*., *ac*.}. The critical temperature can be calculated with the use of Eq. (3).

Let us assume that in high-*T*_*C*_phase *λ*_*opt*_ ≫ *λ*_*ac*_. We also suppose that 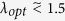. As will be shown below, these conditions are indeed satisfied.

Let us write *T*_*C*_ as 
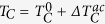
 and assume that 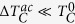. As the first step, let us neglect the contribution of the acoustic phonons. The vaue of 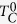 can be obtained from Eq. (3) keeping only the first term on the right-hand side of Eq. (3). As the solution for 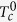, one can use either the McMillan-Dynes expression^37^,^38^ which is valid for 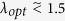, or the close expression, obtained analytically in^34^:


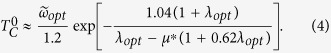


To find a correction 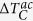 due to the acoustic phonons contribution, consider the full Eq. (3). Substituting the total 
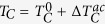
 in the first term on its right-hand side and 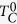 in the second term, we obtain after a calculation (see Supplemental Materials A):


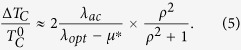


Here 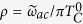. These results can be used to evaluate *T*_*C*_for the cubic high -*T*_*C*_ phase.

The values of the coupling constants and μ* (usually μ* ≈ 0.1 ÷ 0.15) for ordinary superconductors can be determined from tunneling spectroscopy measurements (see, e.g.^32^); tunneling spectroscopy also has been used to study the effect of pressure^39^. Since such measurements have not been performed for sulfur hydride, we deduce the coupling constants *λ*_*opt*_ and *λ*_*ac*_ from several theoretical calculations of *α*^2^(*ω*)*F*(*ω*). Although the theoretical results differ somewhat, they are relatively close. According to^6^,^13^, we estimate *λ*_*opt*_ ≈ 1.5 and *λ*_*ac*_ ≈ 0.5; these values consistent with the above approximations. Using these coupling constants and taking for 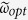 and 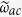 the values 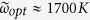 and 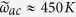 (μ* ≈ 0.14 which is close to that for usual superconductors and was also calculated in^11^), we obtain 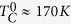 and 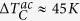, so that in total *T*_*C*_ ≈ 215 *K*, in quite good agreement with *T*_*C*_ ≈ 203 *K* observed in^4^. The main contribution comes from the optical phonons, this confirms the self-consistency of our approach.

The fact that the coupling constant *λ*_*opt*_ in the *cubic* phase is so large is a key ingredient underlying the observed high *T*_*C*_ ≈ 203 *K*. Qualitatively, this is due to the ability of sulfur to retain several hydrogen atoms in its proximity, that is, to the presence of many light ligands near the S atoms.

The method proposed above can be of relevance for other materials as well. A promising example is calcium hydride^40^. The corresponding analysis with the use of our approach will be described elsewhere.

The papers cited above calculate *T*_*C*_ without dividing the phonon spectrum in two parts. As discussed above, the approximation of defining an average 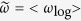 for the entire spectrum is hard to justify. Furthermore, the McMillan-Dynes equation used in these references to calculate *T*_*C*_is not valid for total coupling constant as large as those obtained in^33^,^34^,^35^.

Within our approach, on the other hand, *λ*_*opt*_ is within the range where Eq. (4) is applicable. As for Eq. (3), it allows us to evaluate the relative contribution of the optical and acoustic branches of the phonon spectrum to *T*_*C*_: ~80% is due to the optical phonons and only ~20% is due to the acoustic part.

### Isotope effect

The isotopic dependence of *T*_*C*_ (change upon the substitution of deuterium for hydrogen^2^,^3^,^4^) is of fundamental importance, since it proves (a) that the high *T*_*C*_ state is caused by the electron-phonon interaction and (b) that it is the high frequency hydrogen modes that determine the value of *T*_*C*_. Indeed, the optical modes are mainly due to motion of hydrogen, whereas for the acoustic modes the participation of sulfur ion prevails. Therefore the magnitude of the isotope coefficient reflects indirectly the relative contributions of the each group (optical *vs.* acoustic) into the observed *T*_*C*_.

For the cubic high-*T*_*C*_phase the value of the isotope coefficient (in the harmonic approximation),


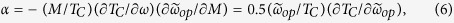


can be evaluated from Eqs (4, 5). After a calculation we obtain:


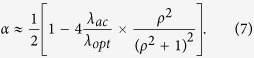


Here 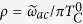. With *λ*_*opt*_ ≈ 1.5, *λ*_*ac*_ ≈ 0.5, 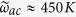 (see the Supplemental Material A) we obtain *α* ≈ 0.35 in good agreement with^4^. Note that the value of *α* can be affected by anharmonicity^12^,^13^ and by the dependence of μ* on 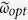, although the last contribution is of the order of (μ*/*λ*_*opt*_)^2^ and is small.

It is noteworthy that the isotope coefficient in the low-*T*_*C*_phase is different. Indeed, according to^6^, the coupling constants for this phase are*λ*_*opt*_ ≈ *λ*_*ac*_ ≈ 1. These values reflect a larger relative contribution of the acoustic modes. In this case 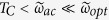 and within the usual BCS logarithmic approximation one can obtain:


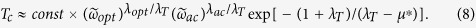


Here *λ*_*T*_ = *λ*_*ac*_ + *λ*_*opt*_; *Z* ≈ (1 + *λ*_*T*_) is included into the exponent^33^,^37^.

With 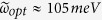 and 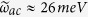 for the low-*T*_*C*_ phase (see^6^) we obtain *T*_*C*_ ≈ 120 *K*.

From Eqs. (6, 8) one finds *α* ≈ 0.25, which is noticeable smaller than for the high-*T*_*C*_phase. Experimentally^4^ the impact of the isotopic substitution in the region of smaller *T*_*C*_ is weaker than in the high-*T*_*C*_ phase, in agreement with our analysis.

Smaller *α* reflects the larger role played by the optical phonons in the *cubic* phase, resulting in its higher *T*_*C*_.

### Phase sequence

The phase diagram of sulfur hydride has been studied with *ab initio* calculations^5^,^6^,^7^,^8^,^9^,^10^,^11^,^12^,^13^,^14^,^15^,^16^,^17^. According to^23^, in the low pressure regime there is a microscopic mixture of phases. The smallness of the entalphy for stochiometric H_2_S-H_3_S boundaries may result in the formation of metastable alloy-like structures containing both components.

A few structures have been identified as the most energetically stable phases. According to^6^, below 100 *GPa* we are dealing with the *Cccm*-structure. On the other side, according to all the relevant publications, at pressures *P* ≥ 200 *GPa* the system forms the body-centered cubic 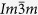 (*Im-3m*) lattice with one entity H_3_S per unit cell. To emphasize, in this pressure range theoretical results^5^,^6^,^7^,^8^,^9^,^10^,^11^,^12^,^13^,^14^,^15^,^16^,^17^ for the electron and phonon spectra differ only in minor details.

At *intermediate* pressures first principle calculations disagree significantly regarding the critical pressure and the symmetry of the phase preceding the *Im-3m* one. According to^6^, the *Im-3m* phase gives way to the phase *R3m* below 180 *GPa*. Both in^11^ and in^6^ the *Cccm* structure remains stable up to *P* = 95 *GPa*.

For the interval *P* = 95 ÷ 150 *GPa* the thermodynamic phase is *R3m* (*β*−*Po*-type), see^11^, but the *Im-3m* lattice sets in at the pressure *P* = 150 *GPa*, instead of ≈180 *GPa* in^6^. The results for the ground state are given in^9^ only for two pressures *P* = 150 *GPa* and *P* = 200 *GPa*. Favorable at *P* = 200 *GPa* is the *Im-3m* structure, but the *R3m* phase prevails at *P* = 150 *GPa*. The last result contradicts^11^, but is in agreement with^6^.

### Thermodynamics of the transition

The rapid growth of *T*_*C*_ in the pressure interval of 125–150 *GPa*^3^,^4^ raises the question of whether this rapid *T*_*C*_-variation is indeed due to a structural phase transition, and if this is the case then what are the two adjacent phases. The *T*_*C*_ data in Fig. 3c of paper^4^ is obtained both while increasing and decreasing the pressure point at the discontinuous transition, although the character of the transition cannot be deduced unambiguously only from the pressure dependence of *T*_*C*_. As shown above, the accuracy of the *ab initio* calculations is insufficient to determine theoretically the precise value of the critical pressure for the transition between the low-*T*_*C*_ and high-*T*_*C*_ phases. One should note, however, for the purpose of determining the order of the transition between the two phases these uncertainties are less relevant than symmetry arguments. To cast the analysis in terms of the Landau theory of the symmetry phase transitions^41^, it is convenient to consider the phase transformations in the reverse order, that is, as a function of *decreasing* pressure.

According to^11^,^12^,^13^, the transition into the *R3m* phase is driven by softening of the sulfur-hydrogen stretching mode. The cubic space group *Im-3m* (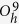) contains *inversion* as one of the symmetry elements. Space group #160 (*R3m*) belongs to the class *C*_3*v*_ for which *inversion* is absent. Hence, the second-order transition between the high-*T*_*C*_ *Im-3m* phase and the phase *R3m* does not contradict to the Landau theory^41^. Note that the notation *R3m* (β *Po*-type) used in^11^ is for the same rhombohedral *R3m* phase as in^12^,^13^.

This specific result^13^ may be sensitive to the calculation details; indeed, for the critical pressure *P*_*cr*_ one finds *P*_*cr*_ = 150 *GPa* in ^11^vs. *P*_*cr*_ = 103 *GPa*^13^. However, with the use of the group-theoretical symmetry analysis, we can prove *rigorously* that the list of the phonon modes available at the center of the Brilloiun Zone (BZ) for the point group *O*_*h*_ = *T*_*d*_ × *C*_*i*_ is comprised of four *odd* three-dimensional irreducible representations (three vector representations *F*_2u_ and one *F*_1u_^42^), so that *any* instability with the zero structural vector would result in the second order transition.

According to^13^, the “imaginary phonon frequencies” appear at several points of the BZ (in the harmonic approximation). Furthermore, to the best of the authors’ understanding, the *first principle* calculations^11^,^12^,^13^ never discussed softening of a phonon frequency 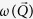 due to its renormalization via the electron-phonon interactions (see^23^,^43^,^44^), and we infer that instabilities with a non-zero structural vector in sulfur hydrides remain unexplored. We mean a structural transition with a change in periodicity or the usual charge density wave (CDW) transition (see in^45^). Note that the problem of the CDW instability with a non-zero structural vector 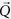 was investigated long ago in transition-metal dichalcogenides with the *incommensurate* and *commensurate* CDW phases separated below the instability point by a first-order phase transition^46^. (The *trigonal R3m* phase with three H_3_S entities per unit cell suggested in^5^ is the example of the commensurate modulated phase).

As pointed out above, the abruptness of the *T*_*C*_-variation^3^,^4^ testifies in favor of a first-order transition. To clarify the issue, X-rays measurements with higher resolution are required.

### Fine bands structure and role of hole-like pockets

The fine structure of the electronic energy spectrum in the high-*T*_*C*_ phase consists of small hole-like pockets at several locations within the BZ, with the Fermi energy on the order 0.5 *eV* ÷ 100 *meV*. As emphasized above, the presence of the pockets seems to be reliably established in the band calculations^6^,^9^,^11^,^12^ (see Fig. 6 in Suppl. to^12^)^17^,^18^,^19^. In addition, tunneling experiments would be able to confirm the existence of small pockets by the observation of the two superconductivity gaps.

However, there is no agreement regarding the importance of the small pockets for superconductivity at the high temperature of *T*_*C*_ ≈ 203 *K* in H_3_S. Since the position of a van Hove singularity peak at the Fermi level appears uncertain, it is worth considering the possibility of superconductivity arising in a pocket without additional special assumptions.

Interaction of carriers on small pockets with high frequency phonons cannot be included into the scheme^26^, as the Migdal parameter^25^ *ω*_*opt*_/*E*_*F*_ for the hydrogen modes is of the order of unity^17^. Leaving aside the vH-peak hypotheses^17^, the temperature *T*_*C*_ for the pairing on a pocket can be estimated in the weak-coupling approximation^47^.

For simplicity, consider carriers on a single pocket with the Fermi energy *E*_*F*_ interacting with one acoustic mode with the frequency *ω*_*ac*_ ≪ *E*_*F*_ and with one optical phonon with a frequency *ω*_*ac*_ ≪ *ω*_*opt*_ (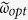 is of the order of *E*_*F*_). Introduce the quantities 
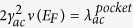
 and 
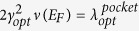
; here *γ*_*opt*_ and *γ*_*ac*_ are the matrix elements of the electron-phonon interactions.

In conventional metals the dimensionless*λ*’s are usually between 1/2 and 1/4. The magnitudes of *γ*_*opt*_ and *γ*_*ac*_ can be assumed to be similar to those in ordinary metals. What makes 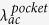 and 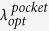 small in the present case is the differences in DOS compared to large Fermi surfaces; then the *T*_*C*_ value possible for hole-like pockets can be evaluated in the weak coupling limit.

The expression for the pairing *T*_*C*_ for a pocket has the form[cf. with Eq. (8)]:


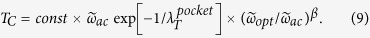


Here 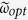 is on the order of *E*_*F*_ and 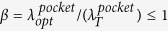. (See in the Supplemental Materials B). Estimating uncertainties in DOS ∝ m* *p*_*F*_ and taking 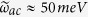 and *ω*_*opt*_/*ω*_*ac*_ ≈ 3 ÷ 4 in Eq. (6) one arrives at a *T*_*C*_ between one and a few tens Kelvin.

In the scenario^17^ a peak in DOS makes the coupling constants 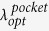 and 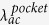 in Eq. (6) large enough to account for the high temperature *T*_*C*_ ≈ 180 ÷ 200 *K* in the cubic phase. The superconducting ordering emerges in the pocket, and induces an order parameter on the large part of the Fermi surface.

As emphasized above, we find this possibility unlikely. A temperature *T*_*C*_ ≈ 215 *K* that was obtained above is close to the values estimated for *T*_*C*_ on the large Fermi surfaces in^5^,^6^,^7^,^8^,^11^,^12^,^13^,^14^,^15^,^16^. In both cases the magnitude of the transition temperature is correct and there is no need for additional mechanisms. Besides, as mentioned above, peaks in DOS are usually located 0.17 ÷ 04 *eV* below the chemical potential.

The above estimates for *T*_C_ in a pocket further confirm the prevailing role of the large part of the Fermi surfaces. We infer, together with^5^,^6^,^7^,^8^,^11^,^12^,^13^,^14^,^15^,^16^, that the superconductivity of hybrid sulfur is driven by phonon-mediated pairing on the broad bands.

One should stress, in addition, that if a van Hove singularity in DOS were assumed to play a leading role, this would result in a change of the prefactor in Eq. (6): 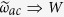 where *W* is the width of the van Hove peak. However, being of the electronic origin the latter cannot depend on the ionic mass, in stark contradiction with the observed isotope effect^2^,^3^,^4^.

### Origin of the *T*_*C*_-maximum in high-*T*_*C*_ phase

The behavior of the temperature of the superconducting transition as a function of pressure is asymmetric with respect to its maximum *T*_*C*,max_≈203 *K* in the high-*T*_*C*_ phase^4^. The rapid *T*_*C*_ decrease at *T*<*T*_*C*,max_ appears consistent with the hypothesis of a *discontinuous* structural first- order transition at *P*_*cr*_ ≈ 123 *GPa*. Additional light on the issue is shed by analyzing the subtle contribution of small pockets.

To describe the major features of the phenomenon, let us consider the two-band model. Then Δ(*ω*_*n*_) and Ξ(*ω*_*n*_) are the two superconductivity order parameters of the pocket and of the broad band, respectively. Assuming that the two bands are weakly coupled, the superconductivity pairing on the pocket change *T*_*C*_ of the whole system only slightly.

Let us, for conciseness, consider only the contribution of the optical phonons. The linear equation for the parameter Ξ(*ω*_*n*_) at *T* = *T*_*C*_ can be written as follows (see in the Supplemental Materials C)


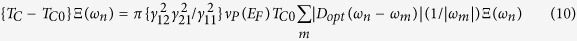


In this equation 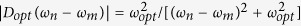, and *γ*_11_ and *γ*_12_ are the matrix elements of the electron-phonon interaction on the large Fermi surface and for electron-phonon scattering between the large and the small Fermi surfaces, respectively (*γ*_12_ ≪ *γ*_11_). (The critical temperature *T*_*C*_ >*T*_*C*0_).

The density of states on the large Fermi surface (LFS) 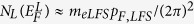 exceeds the one on the pocket 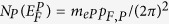 by the factor*p*_*F*,*LFS*_/*p*_*F*,*P*_ 1. Therefore the change in the temperature of the transition *T*_*C*_−*T*_*C*0_ as a function of pressure is simply proportional to the DOS on the pocket. Assume the first- order transition takes place at*P*_*cr*_ ≈ 123 *GPa*. *T*_*C*_ changes from *T*_*C*_ ≈ 100 *K* to *T*_*C*_ ≈ 200 *K*^2^,^3^,^4^ with the pocket emerging simultaneously with the onset of the cubic *Im-3m* phase. ***A** decrease* in *T*_*C*_ after the high-*T*_*C*_ phase onset, according to (10), signifies *shrinking* of the pocket size *p*_*F*,*P*_ with applying higher pressure. This interpretation is in contrast with the scenario^17^ of the pockets developing via the Lifshitz 2.5- topological transition as in that case the pockets sizes would *grow* with pressure.

### Discussion and Summary

From a survey of *ab initio* calculations we conclude that the accuracy of state-of-art first-principles methods is insufficient to identify unambiguously the character of the thermodynamic transition between the high- and low-*T*_*C*_ phase of H_3_S.

We provide arguments that a first-order order phase transition, possibly related to an instability at a finite structural vector, is the most credible concept to account for a step-like increase of *T*_*C*_ at *P*_*cr*_ ≈ 123 *GPa*^4^. We also demonstrate that the decrease in *T*_*C*_ in the high-*T*_*C*_ phase that immediately follows the first-order order transition and the maximum point of *T*_*C*,max_ ≈ 203 *K* signifies that hole-like pockets emerge simultaneously with the transition into the high-*T*_*C*_ phase.

The strong rise of *T*_*C*_ from ≈100 *K* in the low-*T*_*C*_ phase to ≈200 *K* in the high-*T*_*C*_ phase is attributed to *the prevailing* contribution to pairing by high-frequency hydrogen modes over that by the acoustic modes. In the low-*T*_*C*_ phase the two phonons groups contribute to *T*_*C*_ almost equally.

Our analysis points out that methods of calculating *T*_*C*_ based on the McMillan extrapolation, successful for ordinary superconductors, are not applicable to H_3_S because of its complex phonons spectrum comprised of acoustic and several optical hydrogen modes with much higher frequency. The proposed modification for describing pairing on large Fermi surfaces provides realistic values for the temperature of the onset of superconductivity. The calculated isotopic dependence of *T*_*C*_ turns out to be different on the two sides of the transition, in agreement with^3^,^4^.

Comparing the contributions to *T*_*C*_ from the large part of the Fermi surface and from a pocket we conclude that superconductivity in H_3_S is driven by interactions on the former. We point out that the presence of small pockets in the high-*T*_*C*_ phase can be revealed by the detection of *two* superconducting gaps in the tunneling spectra of H_3_S at low temperatures.

The main results can be summarized as follows.

- A first-order phase transition is the most credible concept accounting for the step-like increase of T_C_ at Pcr ≈ 123 GPa observed in^4^.
- The usual methods of calculating T_C_ being inapplicable to H_3_S because of its complex phonons spectrum, we have formulated a modified approach to the full scheme of pairing on large Fermi surfaces. The method is based on separating the contributions of optical and acoustic phonons. It provides realistic values for the superconducting transition temperature and allows us to analyze the relative contributions of the phonon branches (“coupling distribution”).
- The isotope dependence of T_C_ (i.e., its change produced by the deuterium-hydrogen substitution) is evaluated and turns out be different on the two sides of the transition, in agreement with experiments^3^,^4^.
- A microscopic explanation is provided for the unusual behavior of T_C_ in the high-T_C_ phase, namely its decrease with increasing pressure. This irregular behavior of T_C_ above T_C,max_ is ascribed to the presence of small hole-like pockets.
- The contributions to pairing and to the magnitude of T_C_ from the large part of the Fermi surface and that from a pocket are compared. We conclude that superconductivity in H_3_S is driven by pairing on the former.
- The presence of small pockets in the high-T_C_ phase leads to the appearance of two superconducting gaps in the energy spectrum of H_3_S; this can be revealed via tunneling experiments.

## Additional Information

**How to cite this article**: Gor’kov, L. P. and Kresin, V. Z. Pressure and high-T_c_ superconductivity in sulfur hydrides. *Sci. Rep.* **6**, 25608; doi: 10.1038/srep25608 (2016).

## Supplementary Material

Supplementary Information
