# Supplementary material for: Pressure and high-Tc superconductivity in sulfur hydrides
Source: Sci Rep. 2016 May 11;6:25608. doi: 10.1038/srep25608 (PMC4863257; doi:10.1038/srep25608)
Supplement: Supplementary Information [file srep25608-s1.doc]

Supplementary Materials of

*Pressure and high-T_c_ superconductivity in sulfur hydrides*

Lev P. Gor’kov and V. Z. Kresin

**A. Calculation of critical temperature**

In Eq. (3) of the text integrate over$\xi$:

.

Assume that the main contribution to the critical temperature in the high-$T_{C}$phase is due to optical phonons $\lambda_{opt}>>\lambda_{ac}$(, see below). Let$T_{C}\approx T_{C}^{0}+\Delta T_{_{C}}^{ac}$. For$T_{C}^{0}$ use of the McMillan-Dynes expression:

. (A.1)

(This expression valid for ^33,35^ was obtained by including the contribution $Z\simeq1+\lambda$and the fitting to $T_{C}$for Nb (see, e. g., the discussion in ^34^). It appears to be a good description for other strong--‐coupled superconductors.

One can use also the expression, obtained analytically in [24] and valid for :

(A. 1’)

Substituting$T_{C}\approx T_{C}^{0}+\Delta T_{C}$into Eq. (3) one finds$\Delta T_{C}\equiv\Delta T_{C}^{ac}$-the correction due to the acoustic mode. At that one can assume ^35^$\Delta(\omega_{m})=\Delta$. In the equation:

(A.2)

omit the term $\omega_{n}$:

.

In high-$T_{C}$phase$2\pi T_{0}\approx1200K\leq\omega_{opt}$; for an estimate present the sum over *m* as the integral

.

In the second term in (A.2) contribution from $\omega_{n}=\omega_{m}$ compensates$\lambda_{opt}$in$Z\simeq1+\lambda_{opt}$. As, leaving only the next two terms obtain:

$\Delta T_{C}\approx2T_{C}^{0}\frac{\lambda_{ac}}{\lambda_{opt}-\mu^{*}}\times\frac{\nu^{2}}{\nu^{2}+1}$ (A.2)

Here. We use $\alpha^{2}(\omega)F(\omega)$from ^5-9^ to determine $\lambda_{op}$ and$\lambda_{ac}$; from ^5^ $\lambda_{opt}\approx1.5$ and $\lambda_{ac}\approx0.5$.Taking and we obtain$T_{_{c}}^{0}\approx170K$and$\Delta T_{c}^{ac}\approx45K$. $T_{C}\approx215K$.

The estimate is in a rather good agreement with$T_{C}\approx203K$, ($\lambda_{opt}$ and$\lambda_{ac}$ can be measured, at least in principle, with the help of the tunneling spectroscopy (see, e.g. ^29 30^)).

**B. Estimates of temperature of transition on a pocket in the weak –coupling limit**

The equation for the pairing order parameter on the pocket$\Delta(\omega_{n})$at$T=T_{C}$is:

$\Delta(\omega_{n})=-T\sum_{m>0} \int2\nu(E_{F})d\xi\Delta(\omega_{m})[(\omega_{m}^{2}+\xi^{2})]^{-1}\times\left[\gamma_{ac}^{2}D_{ac}(\omega_{ac};\omega_{n}-\omega_{m})+\gamma_{opt}^{2}D_{opt}(\omega_{opt};\omega_{n}-\omega_{m})\right]$. (B.1)

(All notations are as in Eq. (4) of the text). In (B.1) $\nu(E_{F})$is the density of states (DOS) on the pocket Dispersion of the acoustic modes is omitted for brevity. The critical temperature $T_{c}$on the *isolated* pocket is estimated in the logarithmic approximation assuming small$2\gamma_{ac}^{2}\nu(E_{F})=\lambda_{ac}$and$2\gamma_{opt}^{2}\nu(E_{F})=\lambda_{opt}$.

Integration and summation in Eq. (B.1) for contributions from the acoustic and the optical modes are limited by the phonons propagators and $E_{F}$for the characteristic band energy. Introduce $\Delta_{1}$and$\Delta_{2}$ for average values of the superconducting order parameter$\Delta(\omega_{n})$at $\omega_{n}<\omega_{ac}$and $\omega_{ac}<\omega_{n}<\omega_{opt}\sim E_{F}$. Eq. (B.1) reduces to the algebraic system:

$$\Delta_{1}=2\gamma_{ac}^{2}\nu(E_{F})\ln(\omega_{ac}/T_{C})\Delta_{1}+2\gamma_{opt}^{2}\nu(E_{F})[\ln(\omega_{opt}/T_{C})\Delta_{1}+\ln(\omega_{opt}/\omega_{ac})\Delta_{2}]$$

$$\Delta_{2}=2\gamma_{opt}^{2}\nu(E_{F})[\ln(\omega_{opt}/T_{c})\Delta_{1}+\ln(\omega_{opt}/\omega_{ac})\Delta_{2}]$$

The temperature for superconductivity transition$T_{c}$on the *isolated* pocket ($\lambda_{opt}\sim\lambda_{ac}<<1$ and small$\lambda_{opt}\ln(\omega_{opt}/\omega_{ac})$) is:

$T_{C}=const\times\omega_{ac}\exp[-\frac{1}{\lambda_{ac}+\lambda_{opt}}]\times\left(\frac{\omega_{opt}}{\omega_{ac}}\right)^{\beta}$. (B.2)

Here$\omega_{opt}\sim E_{F}$and:

$\beta=\lambda_{opt}/(\lambda_{ac}+\lambda_{opt})\leq1$ . (B.3)

**C. Two band problem**

Let the common approach be employed for the pairing parameter$\Xi(\omega_{n})$ on the large Fermi surface. For simplicity, consider interaction with one optical mode. Weak coupling to the pocket means a *perturbation* of$T_{C}$for the whole system. After simple transformations the resulting equation for the parameter$\Xi(\omega_{n})$ in Eq. (B.1) reads:

$$\Xi(\omega_{n})=\pi T\sum_{m}\gamma_{11}^{2}|\nu_{L}(E_{F})|D_{opt}(\omega_{n}-\omega_{m})|(1/|\omega_{m}|)\Xi(\omega_{n})$$

$+\pi T\sum_{k}\gamma_{12}^{2}|\nu_{P}(E_{F})|D_{opt}(\omega_{n}-\omega_{k})|(1/|\omega_{k}|)\times\pi T\sum_{m}\gamma_{21}^{2}|\nu_{L}(E_{F})|D_{opt}(\omega_{k}-\omega_{m})|(1/|\omega_{m}|)\Xi(\omega_{m})$.

(C.1)

In (C.1) $|D_{opt}(\omega_{n}-\omega_{m})|=\omega_{opt}^{2}/[(\omega_{n}-\omega_{m})^{2}+\omega_{opt}^{2}]$, $\gamma_{11}$ stands for the matrix elements for electron-phonon interaction on the large Fermi surface and$\gamma_{12}$-for the scattering between the large Fermi surface and small pocket($\gamma_{12}<<\gamma_{11}$). Leaving the first term on the right hand side Eq. (C.1) one defines$T_{C0}$-temperature of transition for the large Fermi surface. The second term is the additional contribution from the pocket. At $|T-T_{C0}|<<T_{C0}$ one can rewrite (C.1) as:

$\{T_{C}-T_{C0}\}\Xi(\omega_{n})=\pi\{\gamma_{12}^{2}\gamma_{21}^{2}/\gamma_{11}^{2}\}\nu_{P}(E_{F})T_{C0}\sum_{m}|D_{opt}(\omega_{n}-\omega_{m})|(1/|\omega_{m}|)\Xi(\omega_{n})$. (C.2)
